# The Potential of Poetry in Mental Health Nurse Pre‐registration Education

**DOI:** 10.1111/inm.13509

**Published:** 2025-01-16

**Authors:** Mark Pearson

**Affiliations:** ^1^ School of Health Sciences University of Nottingham Nottingham UK

**Keywords:** health humanities, mental health, nurse education, poetry

## Abstract

This paper examines the potential of poetry as a resource within mental health nurse pre‐registration education. There has long been a debate as to whether the art or the science of nursing should be foregrounded within pre‐registration education, especially in the UK within recent years as the latest Nursing and Midwifery Council's standards of pre‐registration education appear to have shifted the focus towards the acquisition of skills, giving less consideration to the holistic transformatory process of education. The paper uses the conceptualisation of education by Beista, who proposes that education can be considered in relation to the three domains of qualification, socialisation and subjectification. Qualification refers to the acquisition of knowledge and skills, socialisation refers to the process of joining a professional group, and subjectification refers to the development of the individual as a reflective, thoughtful and responsible individual. The paper explores each of these domains in turn and considers how poetry can make a unique contribution in supporting both personal development, and the acquisition of professional knowledge and skills. The reading and writing of poetry can support students to challenge epistemic blinders, enhance their interpersonal skills and develop a more authentic professional identity.

## 1 Introduction

There has long been a debate within nursing as to the intersection between nursing as a science and nursing as an art (Norman and Ryrie 2018) and the way in which these alternative epistemological positions influence the practice and education of nurses (Biley and Galvin 2007). In the field of mental health, the value and therapeutic potential of the arts and humanities are well established (Crawford et al. 2015; Pearson, Rennick‐Egglestone, and Winship 2020; Roberts 2013), but what is perhaps less clear is the role that the arts should play within pre‐registration mental health nurse education.

In the United Kingdom, the Nursing and Midwifery Council introduced new standards of pre‐registration education for all nurses regardless of field of practice in 2018 (NMC 2018). Since the inception of these standards, there has been much debate in relation to their utility (Glasper and Fallon 2021; Leigh and Roberts 2018; Pearson et al. 2024), and importantly for mental health nurses, the impact of these standards in potentially diluting and undermining the specialism of mental health nursing (Connell et al. 2022; Warrender 2022). In relation to the science versus arts debate, these new standards appear to heavily lean towards the science, with a focus on student nurses gaining proficiency in the undertaking of a variety of generic clinical skills.

The acquisition of certain skills is an important element in the training of a mental health nurse, or any other healthcare professional, and consideration must be given to how these can be effectively achieved (McCutcheon et al. 2015). Nevertheless, what is potentially concerning about the new standards is the possibility of nurse education becoming hyper focused on tokenistically achieving generic positivist competencies, and not considering education as being transformatory in nature, based on a process of self‐reflection and emotional intelligence (Freshwater and Stickley 2004). This reflexive transformatory learning process enables students to develop a sense of self that forms the foundation of authentic, compassionate and empathetic practice (Randle 2002).

This paper argues that nurse education needs to be cognisant of the importance of the arts and the way in which art, and particularly poetry, has something unique to offer pre‐registration mental health nurse education, not only in supporting a process of reflection but also in enhancing interpersonal clinical skills. Whilst the precise mechanisms and processes for how the arts can be introduced to mental health nurse education remains rather unclear (Gallagher 2007), this paper uses the three domains of education, proposed by Biesta (2020), to structure an exploration of the ways in which poetry can contribute to mental health pre‐registration education.

## 2 Discussion: Qualification, Socialisation and Subjectification

Biesta (2020) proposed that education can be conceptualised in three domains: qualification, socialisation and subjectification. Qualification refers to the acquisition of knowledge and skills, socialisation refers to the process of joining a professional group, and subjectification refers to the development of the individual as a reflective, thoughtful and responsible individual.

### 2.1 Socialisation

The domain of socialisation is associated with the way in which students are introduced to a particular field, and the vocational and professional norms within that environment (Biesta and van Braak 2020). Fenwick (2008) described learning as both embodied and embedded within social practices and actions. This is important for mental health nurses to consider as the professional social environments they find themselves in, both as students and ultimately as qualified nurses, are complex and historied.

Clinical supervision and self‐reflection has long been a component of nurse education (Howatson‐Jones 2016) and continues to be considered essential in supporting students to explore and understand their experiences of clinical practice (Freshwater 2002; Maplethorpe, Dixon, and Rush 2014). An important element of clinical supervision involves exploring some of the social pressures and factors that not only influence practice but also the construction of professional identity (MacLaren, Stenhouse, and Ritchie 2016). Whilst there are different models and approaches which can be utilised to guide clinical supervision (Blake et al. 2023), these are often applied inconsistently or in some instances, due to bad supervision practices, applied in a way that can become actively harmful to supervisees (Ellis et al. 2017; White and Winstanley 2021).

Jack and Illingworth (2017) write of the need for adopting new aesthetic and personal ways for student nurses to explore their experiences, and the reading and writing of poetry may be able to offer something unique in this process of reflection and supervision. The process of reading and writing poetry is relational in that it provides a space for an individual to consider how they perceive themselves in relation to their environment (Wilkinson 2009). As suggested by Furman, Coyne, and Negi (2008), writing is not only about exploring oneself but also about exploring the world, considering the way in which poetic writing can represent and explore not only the voice of the writer but also the voices of those around them (Wilkinson 2009). In this sense, the process of poetic reflection does not only involve introspection but also consideration of the metanarratives that may orientate social situations and workplace cultures. These may be helpful or unhelpful, compassionate or coercive, but it is the exploration of these which is important, and the writing of poetry, perhaps within a supervision environment, may be able to catalyse this process of exploration. Illingworth and Jack (2018) also suggest that engaging in the reading and writing of reflective poetry, has the potential to support students to build a sense of community and bring students together to discuss the things that might otherwise have remained unspoken.

A fundamental feature of reflective practice and engagement with poetry is the adoption of a pluralistic position; in that individuals can embrace a variety of voices and theories, enabling a permeability of oneself and recognising the potential for flaws in some of the beliefs which we may hold dearest to us (Pollard and Collins 2005; Speare and Henshall 2014). This poetic curiosity and reflexivity can proactively support students to question some of the ‘taken for granted ideas’ (Simmonds and Mozo‐Dutton 2018, 135) that they have encountered in practice. The language used within poetry, rich in symbolism and metaphor, can facilitate multiple readings and potentially elucidate the notion of multiple interpretations emerging from what might, at first consideration, be observed as fixed in its interpretation (Raingruber 2009).

This process may prevent the development of what Hyman (2010, 155) refers to as ‘epistemic blinders’, as when writing autoethnographic poetry (Lahman et al. 2010), students are able to explore areas that may otherwise not be addressed if not prompted (Speare and Henshall 2014). Moreover, this process is both emotional and cognitive, developing critical and reflective thinking whilst also facilitating deeper exploration of personal feelings (Jack and Illingworth 2017).

### 2.2 Qualification

Biesta and van Braak (2020) conceptualise the domain of ‘Qualification’ as the acquisition of knowledge and skills. The specific knowledge and skill requirements of mental health nurse education continue to change and transform, often reflecting the broader changes within the landscape of contemporary healthcare (Doyle 2019). Therefore, much of the discourse is often focused on the notion of competence, which is the ability for an individual to responsibly and autonomously mobilise their knowledge and skills effectively within a practice setting. This definition of competence feels rather detached, as if competence is merely about doing things *to* people, rather than being *with* people.

One of the functions of the humanities, and perhaps poetry specifically, is to support students in becoming competent whilst remaining focused on the personhood and holism of those they are working with in practice. This is not to remove a focus from developing specialist skills, which will be appealing to many students who are drawn to study mental health nursing because they want to learn how to work therapeutically with people with mental health problems. The purpose of the humanities, as suggested by Gordon (2005), is to orientate clinical practice to a position of ‘to cure sometimes, to relieve often, to comfort always’.

Defining what knowledge and skills should be valued within mental health nurse education is complex, as the practice of mental health nursing is often subject to the intersection of multiple knowledge paradigms, all seeking to dominate the orientation of clinical practice (Pearson, Rennick‐Egglestone, and Winship 2023). McKeown et al. (2018, 5) describe the practice of mental health nurses as ‘at its best … intensely relational and humane, drawing heavily on interpersonal resources …’. It is interesting to compare the similarities of this definition to the description given by Viney, Callard, and Woods (2015) when commenting on some of the core features of medical humanities, which they describe as a resistance to biomedical reductionism, a sensitivity to narrative‐based interventions and understandings, and a focus on interpersonal relationships, alongside, and appreciation for the dynamic role of the arts and humanities.

The utility of poetry may be considered to primarily relate to aspects of clinical practice often relegated to the title of ‘soft skills’, a title often used to refer to interpersonal or communication skills (Wald, McFarland, and Markovina 2019). However, the application is much broader than this, for example, Kalra et al. (2016) wrote of using poetry to teach pharmacology, noting that the use of poetry helped students to apply knowledge to real‐life situations. This process of learning can also be understood as a narrative process, underpinned by the development of stories that act as schemas to orientate and support the meaning making and consolidation of new knowledge and experiences (Clark and Rossiter 2008).

In considering learning as a narrative process, consideration must be given to the stories that students are engaging with as part of their learning, as these are the stories that will help them to construct meaning from their clinical experiences. O'Connor (2000) proposed that there are three types of stories, these are stories one likes to tell, stories one must be told and stories that cannot be told. The first category often relates to positive aspects of individual lives and the second to practical issues that need to be addressed within one's lifeworld. However, the third category, the stories which cannot be told, are those often associated with trauma, abuse and shame. These are the stories that no one likes to share but which must be told (O'Connor 2000), and it is these stories that student mental health nurses are often faced with throughout their training.

As poetry often explores the fundamental aspects of being human (Jeffs and Pepper 2005), it has the potential to capture unsayable stories in a way that may represent an important learning resource for students. The below haiku poem by Rollins (2017, 9) represents a unique story, one which it may be impossible to capture other than with a poem. The poem conveys ambivalence, anger and confusion at being resuscitated following a suicide attempt.

It turns out I died
And the bastards brought me back
I wish they hadn't

It is important to acknowledge that poems, such as the one above are highly emotively charged and may provoke significant reactions in those who read them. However, Evans (2021) wrote of the importance of those working in mental health settings willing to be disturbed in the process of engaging meaningfully with a person and their story. Utilising poetry, whether reading or writing, can support students in engaging with distressing and disturbing narratives, as the poem becomes the container for these intense emotions whilst supporting reflection (Davis and Billington 2016) and promoting resiliency (Furman, Coyne, and Negi 2008).

A concern for student mental health nurses is that their work involves much complexity and much uncertainty. However, this complexity and uncertainty also represent rich learning opportunities, and it is important that students do not develop a reductive position in which they merely equate these complexities to attributes of mental illness (Gadsby 2018). The inclusion of the poetry promotes a practice based on curiosity which can support students to move away from a reductive position, towards one that seeks to more effectively tune in to what is being communicated (Pearson, Rennick‐Egglestone, and Winship 2022; Pearson 2024).

### 2.3 Subjectification

Biesta (2020, 93) proposed that this domain is fundamentally interested in freedom, the notion of ‘freedom to act or not to act … exist as a subject in my own life, not as an object of what other people want me to be’. This resonates with the notion of authenticity, the way in which someone can truly be their true self, to be aware of their internal thoughts and states and be able to express these within their lifeworld (Joseph 2016). However, Maté and Maté (2022) described a tension between authenticity and acceptance, which is a tension between living in a truly authentic manner and living in a way that is desirable within a social context.

This tension between authenticity and acceptance is not necessarily new to mental health nurses, with Jackson and Stevenson (2000) describing a key trait of mental health nursing as the construction of the ‘pseudo‐ordinary self’, a combination of what one feels to be their ordinary true self and their conceptualisation of their professional self. Whilst the construction of these different identities may represent growth and development within a field of practice, there is perhaps something pernicious about this process if it results in the disavowal or diminishment of parts of the self. Tischler (2010) described the way in which ‘something is lost’ as medical students progress through their course and despite achieving competencies and acquiring a huge amount of knowledge, the desire that brought them into the profession in the first place becomes diminished.

In reflecting on this sense of loss described by Tischler (2010), it is helpful to consider which stories, especially those narrated by students, are being given room to breathe within preregistration education. Frank (2010, 3) described the way in which ‘… stories breathe life not only into individuals but also into groups that assemble around the telling and believing of certain stories’. Students are often surrounded by discourses of professionalism and professional values (Duphily 2014; O'Connor et al. 2022; Shen et al. 2021); however, there is perhaps a risk that alternative holistic stories become silenced, as students adapt their language to cohere to the requirement of credibility perpetuated by larger professional discourses (Hahessy 2016).

An example of a narrative that transcends the professional discourse towards something more holistic can be observed in a reflection written by Graham (2022), of their personal and professional experiences of the COVID‐19 pandemic. This narrative does not seek to document experiences refracted through the lens of professionalism but rather offers an insight into a human experience of grief and loss. It is these poetic human narratives that can assist students to overcome the isolation that often results from professionalised language and explore the human dimensions of clinical practice (Oyebode 2010). The reading and writing of poetry can help students to reflect on the ‘messy’ (Reed et al. 2012, 12) experiences of being with others, examining both the cognitive and affective components, which might otherwise remain dormant (Mazza 2017).

In considering the conceptualisation of the professional self, Gipps (2022) proposed that those who create a picture of themselves often then start to resemble that picture. Therefore, the place of poetry is to support this conceptualisation of the professional self, to ensure that the picture students are crafting is authentic, holistic, and resonates with the values and beliefs that first attracted them to enter this field of practice. Poems can serve to foster the emergence of a new professional identity whilst still bearing witness to the other multiple stories that construct a student's sense of themselves (Byma and Lycette 2023; White and Epston 1990).

## 3 Conclusion

This paper has used the three domains of education, conceptualised by Biesta (2020) to argue that poetry has a distinct ad meaningful contribution to make to nurse education. Biesta and van Braak (2020, 455) argued that education is an ‘open, semiotic, recursive system that works through communication, interpretation and the thoughtful actions of teachers and students’. In this process of communication and interpretation, poetry can open new opportunities, enabling what was previously unsayable to be articulated (Pearson, Rennick‐Egglestone, and Winship 2022) and providing a space for the exploration of what might have previously been ignored or diminished (Mazza 2017).

## 4 Relevance for Clinical Practice

The reading and writing of poetry have the potential to enrich one's sense of self, facilitating honest contemplation on nursing practice at a deeply human level (Byma and Lycette 2023), and the development of what Chinn et al. (2021, 98) described as ‘personal knowing’. The knowledge and skills of how to be a good mental health nurse are not something that can be transferred as an object from a teacher to a student (Doyle 2019). These are things that are embodied and poetry can perhaps enable these to be embodied at a deeply human level, supporting students to break free of epistemic restrictions and develop as professionals who are fundamentally ‘human beings with feelings and emotions, who are fallible and justly uncertain …’ (von Peter et al. 2021, 4).

## Conflicts of Interest

The author declares no conflicts of interest.

## Data Availability

The author has nothing to report.
